# The construct of cuteness: A validity study for measuring content and evoked emotions on social media

**DOI:** 10.3389/fpsyg.2023.1068373

**Published:** 2023-03-03

**Authors:** Ewa M. Golonka, Kelly M. Jones, Patrick Sheehan, Nick B. Pandža, Susannah B. F. Paletz, C. Anton Rytting, Michael A. Johns

**Affiliations:** ^1^Applied Research Laboratory for Intelligence and Security, University of Maryland, College Park, MD, United States; ^2^Department of Human Development and Quantitative Methodology, University of Maryland, College Park, MD, United States; ^3^College of Information Studies, University of Maryland, College Park, MD, United States; ^4^Institute for Systems Research, University of Maryland, College Park, MD, United States

**Keywords:** cuteness, emotion, kama muta, baby schema, approach motivation, communal sharing, social media, construct validity

## Abstract

Social media users are often exposed to cute content that evokes emotional reactions and influences them to feel or behave certain ways. The cuteness phenomenon in social media has been scarcely studied despite its prevalence and potential to spread quickly and affect large audiences. The main framework for understanding cuteness and emotions related to cuteness outside of social media is baby schema (having juvenile characteristics), which triggers parental instincts. We propose that baby schema is a necessary but not sufficient component of explaining what constitutes cuteness and how people react to it in the social media context. Cute social media content may also have characteristics that evoke approach motivations (a desire to interact with an entity, generally with the expectation of having a positive experience) that can manifest behaviorally in sharing and other prosocial online behaviors. We developed and performed initial validation for measures in social media contexts of: (1) cute attributes that encompass both baby schema and other proposed cuteness characteristics (the Cuteness Attributes Taxonomy, CAT) and (2) the emotional reactions they trigger (Heartwarming Social Media, HSM). We used the Kama Muta Multiplex Scale (KAMMUS Two), as previously validated measure of kama muta (an emotion akin to tenderness; from Sanskrit, “moved by love”) as a measure of emotional reaction to cute stimuli and the dimension Cute Content of the Social Media Emotions Annotation Guide (SMEmo-Cute Content) as a developed measure of gestalt cute content to help validate our newly developed measures. Using 1,875 Polish tweets, our results confirmed that cute social media content predicted a kama muta response, but not all KAMMUS Two subscales were sensitive to cute content, and that the HSM measure was a better indicator of the presence of cute content. Further, the CAT measure is an effective means of categorizing cute attributes of social media content. These results suggest potential differences between in-person, online, and social media experiences evoking cute emotional reactions, and the need for metrics that are developed and validated for use in social media contexts.

## 1. Introduction

A Twitter user says “aww,” and clicks the “retweet” button on a video of a box of kittens. A child sees a puppy in a window and begs their parents to adopt it. A jogger smiles when passing a baby in a park. Cuteness comes in many forms: animals, people, objects, noises, and scenes. They affect us in many, sometimes surprising ways. They serve as stimuli to evoke different physiological, behavioral, and emotional reactions in people who experience them (Glocker et al., 2009a, 2009b; Kringelbach et al., 2016; Steinnes et al., 2019). Understanding what attributes make an entity cute, what emotion cuteness evokes, as well as how to objectively measure those attributes and emotions are all important, because cuteness can influence people, and challenging, because perception of cuteness is commonly based on a subjective judgment (though there is agreement on some common characteristics of cuteness, see Nittono et al., 2021).

Compounding the challenge is that cute stimuli evoke in people an emotion that is positively correlated with–but not identical to–well-researched emotions such as love and happiness.^1^ It is a distinct emotion that fits the standard definition criteria for emotions that include “appraisals, experiences, expressive behavior, physiological response, influences upon ensuing thought and action, and language-based representations of these unfolding processes” (Cowen and Keltner, 2021, p. 125). However, to date, very little research exists concerning this emotion that is evoked by cute content. The psychology of emotions has been criticized for focusing too much on emotion terms that come from English (Ortony, 2022), and in fact, no single term exists in English for this emotional response, although it is sometimes referred to as “cuteness response” (Sherman and Haidt, 2011), “cute-emotion”, or represented by the vocalization “aww” (Buckley, 2016). Steinnes et al. (2019) linked the emotion evoked by exposure to cute content to the emotion of kama muta (“moved by love;” Fiske et al., 2017; Zickfeld et al., 2019).

However, the available research on cuteness attributes and the emotion evoked by cute content has been conducted in environments other than social media, even though the internet and social media are common places where people encounter cute content. As emotions arise from interpretations and evaluations of the situation (Barrett et al., 2007), it is useful to evaluate them within the specific context in which an emotion is evoked. Given how fast cute content can spread (e.g., propagate across social media), it is important to understand the power of this phenomenon and how it can affect social media audiences, which can be done in a range of ways: through inducing positive emotions and making people feel good (Lien and Wu, 2021), convincing people to buy commercial products (Lu et al., 2021), change behaviors (McBride and Ball, 2022), or even manipulating, dividing, or disrupting populations (e.g., Farwell, 2014; Whitehead, 2016).

Our research seeks to operationalize two aspects of cuteness on social media for future quantitative research, such as on the relationship between cute social media stimuli and online behaviors. Specifically, our goals are to propose and accrue initial validity evidence for (1) a measure of the characteristics present in cute content on social media (Cuteness Attributes Taxonomy, CAT), and (2) a measure of the emotional reaction to cute stimuli that occurs in social media (Heartwarming Social Media, HSM). We used KAMMUS Two, a validated measure of the kama muta emotional reaction (Zickfeld et al., 2019), as a comparison point for our newly developed cute emotion measure, though with the recognition that it was developed for off-line experiences. We also compared our new measures to the gestalt Cute/Kama Muta dimension within the novel Social Media Emotions Annotation Guide (Paletz, 2018; Paletz et al., 2020, 2022).

### 1.1. Cuteness as a universal construct

The perception of cuteness is generally universal across cultures (Nittono et al., 2021); however, there are differences in the way the concept itself has originated and might be understood. In English, the meaning of “cute” (a shortened form of “acute”) has evolved from the original “shrewd,” “clever” or “quick-witted” in the 18th and 19th centuries, to a more esthetic “attractive” or “charming” as well as being associated with small size in the 20th century (Halperin, 2012; Waldman, 2015). Major dictionaries list both senses: “clever or shrewd” as well as “attractive or pretty especially in a childish, youthful, or delicate way” (Merriam-Webster, 2023). More recently, this definition has been expanded to include social engagement: “Cuteness is an appeal to others: an invitation to engage in social behaviors including companionship, cooperative action/play and communication through emotional reactivity” (Dale, 2016, p. 8). In this study, we recognize both aspects and thus define cuteness as having some combination of the following characteristics: being perceived as having juvenile characteristics, being adorable, and/or evoking positive, prosocial emotions and approach motivations. We do not include the concepts of sexy-or erotic-cute and uncanny-cute (May, 2019).

The concept of cuteness exists in multiple languages and cultures even if there is no lexical equivalent for it, as for example, in Polish, where either an English word “cute” or vernacular words for “sweet/sweetie” (*słodki/słodziak*), “adorable” (*uroczy*), “delightful” (*rozkoszny*), or “cuddly” (*milutki*) have been used. In Japan, cuteness is often associated with the *kawaii* culture, whose origins go back to the 10th century, although they are not identical. The meaning of kawaii as an affective state has evolved over the years from “ashamed” and “pitiable” to the contemporary “a positive emotion related to the social motivation for engaging and staying with preferable persons and objects, which is typically observed in affection toward babies and infants, but not limited to them” (Nittono, 2016, p. 91). Kawaii as an artistic and cultural style remains an essential part of Japanese culture and has a huge impact on the toy, fashion, art, and film industries, especially, but not exclusively, targeting girls and young women (Marcus et al., 2017).

Even though the concept of cuteness is universal, not all people within the same cultures and languages perceive cuteness the same way. Individual differences in perceiving cuteness usually pertain to gender, showing that women are more sensitive to cuteness than men (studies conducted in Europe and United States; Sprengelmeyer et al., 2009; Lobmaier et al., 2010) or age (Sprengelmeyer et al., 2009). However, Takamatsu (2020), whose study was conducted in Japan, found no gender differences in perceiving cuteness among parents with children under the age of six. Individuals may also vary in their responsiveness (sensitivity) to cuteness, commonly measured *via* self-report scales (Lehmann et al., 2013; Takamatsu, 2020). Despite individual differences in perceptions of or responsiveness to cuteness, there are some generally agreed upon attributes or characteristics that define “cuteness” across cultures and languages, as discussed in the next section.

### 1.2. Attributes of cuteness content

The modern concept of cuteness has been influenced by the work of an Austrian ethologist, Konrad Lorenz, who coined the term *Kindchenschema* (baby schema) to refer to child-like features such as small body size with a large head, large eyes, round cheeks, small limbs, plump body shape, soft body surface, as well as being helpless and having clumsy movements. Based on his work on animals, Lorenz postulated that these features evoke caretaking behaviors from adults which increases the chance of survival of the young creatures (Lorenz, 1943). Recent research studies that experimentally manipulated human and animal faces using computer graphic techniques support Lorenz’s argument and show that the presence of characteristics of baby schema in humans and animals is believed to contribute to the perception of them as being cute (Alley, 1981; Glocker et al., 2009a; Little, 2012; Yao et al., 2022). Based on this research, datasets of cute infant faces have been developed and validated (e.g., Japanese Cute Infant Face Dataset; JCIF: a dataset of 50 images of 6-month-old Japanese infants; Nittono et al., 2022).

Metrics for pinpointing baby-like characteristics of human faces and measuring the level of cuteness in human faces utilize various mathematical techniques such as models that allow altering the parameters of human or animal faces (Jones, 1995; Glocker et al., 2009a; Borgi et al., 2014), models to determine the level of cuteness in human faces (Wang et al., 2015), and models for approximating human perception of cuteness (Makula et al., 2017). Despite some of these models having relatively low accuracy rates (e.g., Makula et al., 2017), research investigating the level of cuteness in images of faces with altered attributes is becoming increasingly popular and its findings have been used to inform product design and consumer research, where specific parameters of cuteness may affect product popularity (e.g., Marcus et al., 2017). Metrics for judging the level of cuteness in images of human and animal faces include self-report scales (e.g., Nittono et al., 2022; Zhou et al., 2022) and discrimination tasks (Nittono et al., 2022). Kindchenschema can even be extended to inanimate, abstract objects such as geometric shapes. Cho et al. (2020) had participants modify rectangles along five parameters (size, color, angle, height-width ratio, and roundness) to produce “cute” rectangles, and as hypothesized those rectangles determined to be “cute” conformed to the Kindchenschema characteristics (such as rounder edges and smaller size).

But are Kindchenschema characteristics a necessary condition for an object to be perceived as cute? Even the presence of a baby in the image is not necessarily a guarantee that viewers will judge it cute. Studies on altering baby faces or judging more-and less-cute baby faces showed that while baby schema was present in all images, participants judged some baby faces, such as those with positive expressions (Hildebrandt, 1983) as more cute than the others. Similarly, Nittono (2016) found that baby schema is only one of several attributes associated with kawaii or cuteness, alongside attributes such as smiles, having rounded shapes, and specific colors. Importantly, these other, non-baby-schema-related cuteness characteristics produce comparable positive emotions and approach motivation (Nittono and Ihara, 2017). Approach motivation is the desire to interact with an object, person, or situation, generally (though not always) one that is expected to produce pleasurable or enjoyable emotions or experiences (Harmon-Jones et al., 2013). The “approach motivation” is an alternative or supplementary explanation for the reactions to cuteness stimuli to the Kindchenschema explanation (which posits that cuteness evokes caretaking behaviors). Thus, it is potentially a crucial explanation for reactions to cute images in social media, an environment in which one (generally) cannot engage in caretaking behaviors toward the cute image; instead, one can approach, interact with, view again, or share the cute image.

In the online realm, the literature on user experiences or user interfaces, in addition to baby schema attributes, lists multiple other characteristics that make computer-based products cute. A cuteness taxonomy developed by Marcus et al. (2017) includes the following elements of cuteness: media (e.g., emojis, emoticons), appearances (e.g., colors, shapes, anthropomorphism), sounds (e.g., high-pitched, baby-like), language (e.g., specific vocabulary), and behavior (e.g., gestures or posture that make the entity be perceived as cute). One additional linguistic attribute of cuteness is the frequent use of diminutives in the online contexts, e.g., in Chinese social media (Li, 2021). While several emotional content annotation guides exist, most focus on a small set of 6–9 emotions, and to our knowledge, none cover cuteness or kama muta (see Bostan and Klinger, 2018 for a review) with the exception of the Social Media Emotions Annotation Guide (SMEmo Guide) 3.32, originally developed as part of an effort to analyze 20+ emotions in social media which includes cute content (Paletz, 2018; Paletz et al., 2020; version 4.0 Paletz et al., 2022; see use in Murauskaite et al., n.d.).^2^ The SMEmo captures a broader array of emotions as have been detected in recent research (e.g., Cowen and Keltner, 2021). Our proposed Cuteness Attributes Taxonomy (CAT) incorporates these multiple possible aspects of cuteness characteristics, which draw on both the traditional Kindchenschema and the broader attributes of cuteness which suggest an approach motivation.

### 1.3. The “cute emotion” reaction

Cute stimuli—entities displaying attributes perceived as cute—trigger positive, prosocial emotional reactions in people. Existing research studies on the reactions triggered by cute stimuli generally fall into two categories of explaining the emergence of the emotion: a traditional view supporting the claim that cuteness evokes parental instincts (Lorenz, 1943) or a modern view stating that cuteness evokes social behaviors and communal sharing (Dale, 2016; Steinnes et al., 2019).

#### 1.3.1. Cuteness evokes parental instincts

Supporting the traditional view that cuteness evokes parental instincts (Lorenz, 1943), one line of research shows that cute creatures displaying baby schema characteristics trigger nurturing and helping behaviors in adults (Glocker et al., 2009a), even toward creatures that are not baby humans (Golle et al., 2013). Some early neuroscientific evidence supporting Lorenz’s baby schema claim came from a study by Kringelbach et al. (2008), who observed changes in the orbitofrontal cortex when study participants were looking at pictures of infants and adults. In a neuroimaging study of 16 nulliparous women (women who have never given birth), baby schema activated the accumbens nucleus, a brain area that plays a role in cognitive processing of rewards and motivation, which prompted the researchers to link baby schema to caregiving desires (Glocker et al., 2009b). Further, similar (though not exactly identical) brain regions are activated when mothers view their child and when they view their pet, in addition to expressing similar positive emotions (Stoeckel et al., 2014). Pet owners also better discriminate infantile facial characteristics, and may be more sensitive to cuteness (Borgi et al., 2014).

Studies on physical carefulness show correlations between viewing images of cute animals and improved performance on fine-motor dexterity and non-motor tasks, which researchers stipulate to be an adaptation facilitating caregiving behaviors (Sherman et al., 2009; Nittono et al., 2012). The level of responsiveness to cuteness stimuli, as measured by a 15-item Cuteness Responsiveness scale, has been found to be a motivator for caretaking behaviors among parents of children under the age of six: Parents with lower responsiveness to cuteness tended to be more approving of corporal punishment (Takamatsu, 2020). A related view posits that people are attracted to cute stimuli. This phenomenon has been demonstrated in several psychological studies that show visual preferences for baby vs. adult faces and cute vs. less-cute baby faces (Hahn et al., 2013; Sprengelmeyer et al., 2013). Adults are also able to detect very small differences in human faces that were altered for cuteness (Sprengelmeyer et al., 2009; Lobmaier et al., 2010).

However, not all research supports the relationship between cuteness and caregiving instincts. Nenkov and Scott (2014) found that after exposure to cute products, consumers tended to choose more indulgent self-reward options. They argued that cute stimuli primed mental representations of fun and resulted in indulgent consumption, the opposite reaction to caregiving, which manifested itself in a much more careful behavior. Other research suggests that men show attraction to women with neotenous (juvenile) facial proportions such as large eyes, small noses, and full lips (Jones, 1995)—a finding that seems contradictory to the parental instinct hypothesis.

Cute aggression, defined as “the urge some people get to squeeze, crush, or bite cute things, albeit without any desire to cause harm” (Stavropoulos and Alba, 2018, p. 2), is an example of a multi-layered response to the “cute emotion,” showing both caregiving and non-caregiving responses to cute stimuli, e.g., care and aggression (Aragón et al., 2015). People wanting to squeeze or bite Baby Yoda illustrates the cute aggression phenomenon (Chamary, 2020), where wanting to squeeze a cute baby can be seen as a caretaking response, while biting a cute baby cannot. Stavropoulos and Alba (2018) used event-related potentials to measure neural components related to emotional salience (N200), reward anticipation (SPN), and reward processing (RewP) to study neural correlates of cute aggression in adults (*N* = 54). They observed dimorphous tendencies in expressing positive emotions and concluded that the feelings of cute aggression relate to both feeling overwhelmed by positive emotions and feelings of caretaking.

#### 1.3.2. Cuteness evokes social behaviors and communal sharing

Rather than focusing on parental or nurturing instincts, recent work emphasizes reactions related to social engagement when experiencing cute stimuli. For instance, cute stimuli have been used to elicit moral emotions that trigger social engagement such as play and other affiliative interactions, including the desire to connect with others (Sherman and Haidt, 2011). Contrary to love, compassion, or gratitude, a cuteness response is often directed toward inanimate objects, which explains anthropomorphic tendencies in the toy industry, motion pictures, and marketing (Sherman and Haidt, 2011). Sherman and Haidt (2011) also suggest that “cuteness” is a direct opposite to the emotion of disgust, alongside emotions such as love, gratitude, or compassion. In extreme cases of disgust, the person or object is “pushed beyond the protection of the moral circle” where harm is not prohibited (p. 247). Cuteness, on the other hand, confers positive social values, indicating something to be protected, valued, included, or shared with others as a means of demonstrating those qualities to them. In other words, one might share a cute picture as an invitation to engage in social interaction or as a demonstration of in-group affiliation.

Steinnes et al. (2019) linked the emotion evoked by exposure to cute content to *kama muta,* a Sanskrit term that can be translated to English as “moved by love.”^3^ It refers to the feeling people sometimes have when communal sharing relationships intensify. Communal sharing is a mental representation of a particular aspect of social relations among humans such as familial or friendship bonds, and is contrasted with other models of social relations, such as hierarchical relationships or carefully balanced equal reciprocal relationships (Relational Models Theory; Fiske, 1992). In communal sharing, people do not keep track of what is given or received. In such contexts, English speakers describe kama muta as being touched or heart-warmed, but may also use labels such as nostalgia, patriotism, or rapture (Fiske et al., 2017). Findings obtained by Zickfeld et al. (2019) indicate that kama muta is related to constructs such as empathic concern or nostalgia, but it is a distinct emotion, different from sadness, awe, and amusement, even though all four trigger similar physiological reactions. The authors posit that kama muta can occur either simultaneously with or immediately after other emotions.

Kama muta is an intense, positive feeling that people often wish to share with one another. People can feel kama muta on various occasions; for example, when experiencing romantic love, family relationships, friendship, team spirit, when watching a movie, reading a poem, or when being exposed to an image of puppies or kittens. When people feel kama muta, they become more dedicated and committed to communal sharing; they may experience a special bond with someone, sense of connection, belonging, or a feeling of being appreciated, wanted, or needed (Zickfeld et al., 2019). The feeling of kama muta is usually accompanied by some physical sensations (e.g., moist eyes), physical reactions (e.g., putting hands on a chest), or linguistic labels (e.g., heartwarming).

The most widely used measure of kama muta is the Kama Muta Multiplex Scale (KAMMUS; Fiske et al., 2017; Zickfeld et al., 2019). In a KAMMUS validation study, Zickfeld et al. (2019) recommended the use of KAMMUS Two, a 28-item measure comprised of five subscales (see section 2.3> for description). The kama muta construct has been found to be associated with similar emotional reactions in multiple countries and across multiple languages; however, some variations were found as well (Seibt et al., 2018; Zickfeld et al., 2019). Steinnes et al. (2019) investigated the effect of kama muta on participants (*N* = 356) exposed to cute videos and found that videos of cute targets evoked more kama muta than videos of less-cute targets, as measured by KAMMUS (versions 1.8 and 2.0). They also found that videos where cute targets interacted evoked more kama muta than those where targets were not interacting.

### 1.4. Overview of the current study

As established above, the construct of cuteness is relevant to a wide array of research interests, including persuasion, message propagation, influence, and emotions; however, no comprehensive or unifying measures of cute content or evoked emotion exist, particularly for social media. We present initial validity evidence of two new measures to fill this existing gap in the literature: (1) a measure of cuteness attributes (CAT) specifically designed for social media, using our previously developed gestalt measure of cuteness in the content of a social media post (SMEmo-Cute Content) as a comparison measure; and (2) the Heartwarming Social Media (HSM) scale, as a measure of the emotion triggered by cute stimuli, using the KAMMUS Two as a comparison measure (Zickfeld et al., 2019). We answer the following research questions:

*RQ1*: What is the most parsimonious way to characterize the relationships between possible attributes of cute social media content in the proposed Cuteness Attributes Taxonomy (CAT)?

*RQ2*: Do attributes of cute content (as measured by the CAT) predict experiences of ‘cute’ emotional reaction, as measured by both the established metric KAMMUS Two and the proposed metric Heartwarming Social Media (HSM)? Does the HSM provide added value alongside the KAMMUS Two in this context?

*RQ3*: Do attributes of cute content (CAT) predict gestalt cute social media content (indexed by SMEmo-Cute Content)? In the relationship between cute emotional reactions and cute social media content, does the newly developed Heartwarming Social Media (HSM) scale contribute above and beyond the established kama muta emotions (KAMMUS Two)?

## 2. Materials and methods

### 2.1. Data collection and processing

We conducted our study in Polish social media and language. Because there is no exact Polish equivalent of the word “cute,” we compiled a list of Polish words commonly used in this sense. First, five native speakers of Polish independently generated lists of words that they believed might be present in Polish tweets with cute content. The combined list contained 60 items, of which seven were generated by more than two speakers. Most items were native Polish words, but the list also contained borrowings from English (cute, słit/sweet, bejbi/baby) and Japanese (kawaii) as well as onomatopoetic strings (e.g., uwu, owo, aww) and letter symbolism (e.g., XOXO). The combined list of 60 words was expanded to include relevant inflectional forms^4^ of the inflected (Polish) nouns and adjectives; this process yielded 231 unique keyword strings.

Purposive sampling for cute annotation was performed in multiple stages. In Stage 1, we used a corpus of 762,416 tweets originally collected for another study involving Polish social media over a period from July 2009 to January 2020, collected from 303 Twitter accounts identified as influential sociopolitical groups and individuals in Poland.^5^ We searched this corpus for cute content using the generated “cute keywords,” which yielded 201 tweets that were later annotated. Due to the low yield of cute content in the corpus collected in Stage 1, we collected a new corpus of tweets for a more thorough analysis of cute content posted in Polish Twitter. In Stage 2, a new corpus of 19,592,791 tweets was pulled from Twitter using the original, expanded set of 231 keywords (January 2015 through July 2020) across everyone using Twitter in Poland.^6^ From this new corpus, we randomly sampled one tweet per account containing at least two distinct keywords from the list (for 2015) and at least two distinct keywords *and* some embedded non-text media (e.g., an image or a video) for other years. This process yielded 6,035 tweets, of which 667 were randomly selected for annotation (the original aim was 600 tweets; 667 is the number of tweets annotators were able to code in the time they had available for this task). To increase the diversity of topics within the annotation, in Stage 3, we further refined the keywords to include 40 baby animal names (e.g., piglet, bunny) and seven words referring to human babies. We then sampled one tweet per Twitter account, yielding 3,302 tweets. From tweets sampled in this stage, 743 were randomly selected for annotation. Finally, in Stage 4, in order to increase the range or degree of cute content across the annotated set, we sampled 2,363 tweets from the same accounts but with no keyword list. From tweets sampled in this stage, 280 were selected for annotation.

Altogether, 11,901 tweets were sampled in four stages, of which 1,891 were selected for annotation. All tweets sampled in Stage 1 were annotated, while tweets from Stages 2–4 were randomly selected for annotation. Sixteen tweets were unavailable at the time of annotation, resulting in 1,875 tweets annotated. See Supplementary material for details on data collection and processing.

Utilizing this multi-step methodology to collect and process data allowed us to improve the quality of our sample with each stage and in the end collect a rich sample of posts on diverse topics and containing a range of cute content (on the 0–100 scale) to test new instruments on. However, we acknowledge that this procedure could affect generalizability of our findings to other contexts (see section 4.3).

### 2.2. Annotators

We trained nine Polish annotators (8 women) between the ages of 22 and 27 (*M* = 23.5; *SD* = 1.51) to annotate Twitter posts from Poland. They all lived in Poland and their nationalities were Polish. The annotators were thus native speakers and cultural experts in the content of the tweets. They were psychology students at a large public university in Poland (2) or recent graduates from the same program (7). The annotators underwent extensive training, including discussions of how to identify emotions in their own national social media, how to use the codebooks, as well as multiple hours of practice on training data. Because these annotators had worked on a broader project on emotions in social media (Paletz et al., 2020), they were able to distinguish nuances between their reactions and post content in different positive emotions (e.g., love, happiness, admiration).

### 2.3. Instruments

#### 2.3.1. Social media emotions annotation guide

For gestalt/global assessment of cute content in social media posts, we used the Social media emotions annotation guide (SMEmo) Guide version 3.32 (Paletz, 2018; Paletz et al., 2020).^7^ The SMEmo entailed having annotators assess each social media post for both the content of the post (Cute Content), which includes both the emotion and the stimuli within post, and separately the annotators’ personal reactions. Cute content (SMEmo-Cute Content; Supplementary material) was judged globally and heuristically on a 0 (none) to 100 scale by the presence of the following attributes: (a) sensory/appearance characteristics (baby schema, e.g., small size, round face, big eyes, chubby cheeks), (b) personality characteristics/behavior (e.g., softness, vulnerability, playfulness, clumsiness), and (c) cuteness by contrast (by contrasting opposing attributes, e.g., large dog vs. small dog). In addition, each post’s Cute Content was judged based on the presence of the emotion of reacting to cute stimuli displayed in the message either by the author of the message or others described in the message. For each tweet, annotators independently assigned a value between 0 and 100, where 0 indicated no cute content and 100 indicated very explicit, intense, and frequent cute content. Different fields have explored using different rating scales (e.g., Clemente et al., 2019, for medicine; Dawes, 2008, in marketing; and Preston and Colman, 2000 for psychology). These studies show little differences in psychometric or response properties with between 7 and 101 response options. When the SMEmo was first created, the researchers tested 3-, 5-, and 7-point scales and found that they were not granular enough, nor intuitive to use for assessing posts. The 0–100 scale was based on previous literature on emotion annotation (Strapparava and Mihalcea, 2007), its intuitiveness to annotators, its ability to discriminate between small differences, and its usefulness when providing and comparing ratings for multiple constructs. Empirically, in our study, we found that the annotators did indeed make use of the full range of the scale (0 to 100) and took advantage of the granularity (e.g., using 5, 8, 80, 100, etc.). Consensus meetings were held to come up with one final consensus value for each tweet. This agreed-upon value was used in the analyses, while the individual annotator values were used to calculate inter-annotator reliability.^8^

#### 2.3.2. Cuteness attributes taxonomy

To investigate the specific characteristics of cute content in social media, we developed the Cuteness Attributes Taxonomy (CAT; Supplementary material). The first author wrote the original CAT guide that consisted of 13 items to measure attributes of cuteness in social media. The guide was constructed based on a review of literature with the goal to capture the multimodality of social media messages, e.g., accounting for text, still images, video, and audio, as well as linguistic features specific to online communication. Using this guide, the first author and nine annotators (all native speakers of Polish) first annotated together 16 tweets, in discussion, next annotated additional 40 tweets individually, then met twice for discussion. This process resulted in multiple adjustments to the definitions in the CAT guide. For example, a definition of cute interaction was expanded to specify that cute interaction can be either mutual or reciprocal and either physical or verbal. The group also prepared a list of cute emoji and emoticons that was used as a reference by annotators. During this process, five additional items inspired by the kawaii and cuteness framework (Nittono, 2016) and moral emotions research were tested. Discussions revealed that three of them (smile, roundness, and color) did not yield any cute content in our Twitter dataset and therefore were not included. The group decided to proceed with two other items (anthropomorphism and wholesome behavior), which increased the number of CAT items to 15. The final items in this codebook fall into four categories of codes related to (1) the cute object and its characteristics (whether human, animal, thing, more than one cute object are present, whether the object displays baby schema or anthropomorphic features), and (whether cuteness was conveyed by contrast); (2) visual cues (whether there is an image, emoji, or emoticon in the post); (3) cute behavior (whether there is cute or wholesome behavior or interaction visible); and (4) linguistic features (whether diminutives or cute talk are used). These categories derive from previous research and draw on various perspectives: (1) traditional Kindchenschema research (for baby schema; e.g., Glocker et al., 2009a); (2) user experience research (for the use of emoji, emoticons, anthropomorphism, cute talk, cute behavior, and cuteness by contrast; Marcus et al., 2017); (3) moral emotion research (for interaction and wholesome behavior; e.g., Sherman and Haidt, 2011; Nittono and Ihara, 2017); and communication studies (for an image, the presence of a cute human, animal, or thing, e.g., Lien and Wu, 2021).

We used a binary code (0 = no, 1 = yes) to indicate discrete cute content such as whether the tweet contained a cute human, animal, thing, emoji, emoticon, image, diminutive word form, anthropomorphism, and whether there was more than one cute target item in the tweet, e.g., three bunnies, a cute hat on a cute puppy (each of the above categories were coded 0 or 1). The binary distinction was a natural choice for the discrete items as in these cases we were interested in the presence or absence of a particular attribute, e.g., whether the post included a cute image. In addition, we used a 0–100 scale to indicate continuous cute schemas such as to what extent there was cute talk, interaction, behavior, wholesome behavior or baby schema depicted in the tweet and to what extent cuteness was conveyed by contrast. As with SMEmo, using a 0 to 100 scale for continuous cute schemas allowed for a more granular annotation of how much of, for example, cute interaction was in the post vs. whether or not cute interaction was present in the post.

#### 2.3.3. KAMMUS two

To assess the experience of the kama muta emotion after being exposed to social media content, we used KAMMUS Two, developed and validated by Zickfeld et al. (2019); an earlier version was used to assess the level of kama muta when looking at images of cute and less-cute animals (Steinnes et al., 2019). KAMMUS Two incorporates 28 items grouped into five subscales: (1) sensations and signs (12 items, e.g., experiencing moist eyes, goosebumps, chills); (2) communal sharing appraisal (8 items, e.g., either feeling or witnessing a sense of closeness, special bond); (3) motivations for communal sharing (4 items, e.g., wanting to hug someone or do something extra-nice for someone); (4) valence (1 item, i.e., having positive feelings); and (5) labels (3 items; wanting to describe the experience as heartwarming, moved, or touched). The responses on this scale were assessed on a scale from 0 to 6, where 0 meant *definitely no* and 6 meant *definitely yes*.^9^

#### 2.3.4. Heartwarming social media scale

To address user reactions to cute stimuli on social media, we created a 10-item Heartwarming Social Media Scale (HSM; Supplementary material). Because to our knowledge reactions to cute stimuli on social media have not been previously studied, all items in this instrument are exploratory in nature. The first author wrote the original items for this scale, then discussed and practiced using them on 16 tweets with nine Polish annotators. The goal of this practice was for annotators to understand the scale the same way, and not to reach a consensus. Annotators were reminded that this instrument measures their own reactions to cute stimuli in Polish tweets, regardless of the content of the tweets. Next, annotators continued practicing using the scale by individually annotating 40 tweets, after which another discussion was held when minimal revisions to the items were made. The final 10 items represent detectable reactions of social media users after being exposed to cute content on Twitter. Some items target common online behaviors, such as wanting to have the experience again,^10^ wanting to tell someone about the experience,^11^ and wanting to share this experience with others.^12^ Other items target behavioral reactions to cute stimuli that can be experienced *via* social media. Specifically, we incorporated one item (saying “aww”) that was included in earlier versions of KAMMUS [e.g., version 1.8 and 2.0 used by Steinnes et al., 2019], but was excluded from KAMMUS Two that was used in this study. Saying “aww” is especially relevant to experiencing heartwarming feeling when exposed to cute content on social media as this is a common vocalization when seeing a cute baby or animal (Buckley, 2016). The remaining items in this category include wanting to interact with the depicted person, animal, or object as well as wanting to describe the experience using specific vocabulary (cute, sweet, adorable, delightful, and wholesome). Because the intent of the HSM scale is to measure to what extent the respondents agree or disagree with particular statements about their emotional reactions to cute stimuli, as with the KAMMUS Two, the responses on this scale were assessed on a scale from 0 (definitely no) to 6 (definitely yes).

### 2.4. Procedures

Nine annotators were trained in the SMEmo-Cute and CAT guides as well as the KAMMUS Two and HSM instruments. During annotation, all content in the tweet was assessed and taken into consideration, including text, images, emoji, audio, video, and any preview images of links. For each tweet, annotators first separately and independently assessed the overall value for cute content in that tweet using the SMEmo-Cute Content measure (Paletz et al., 2020). Next, annotators independently assessed the cuteness attributes for that tweet, using the CAT measure. Finally, they assessed the level of the kama muta emotion a given tweet evokes using KAMMUS Two and their own reactions to the content of the tweets using the HSM scale.

Annotators’ individual CAT values and the SMEmo-Cute Content values were entered into a single document and compared. For items on a 0–100 scale, we used two explicit heuristics for whether to discuss discrepancies between annotators. First, if any annotator gave a tweet a zero value and any other annotator gave it a non-zero value, then the team needed to discuss the difference in assessments until a consensus emerged. Second, if on scale items, there was a difference of more than 20 points (out of 100) between any of the assigned codes, then the annotators had to discuss the discrepancy until there was consensus. Otherwise—in cases of only a 20-point difference or less between assigned codes and one of those codes did not equal zero—the annotators averaged their assessments without discussion. On binary items, all discrepancies needed to be discussed. In cases when during a consensus meeting annotators found it difficult to reach agreement, a fourth judge helped resolve any discrepancies. Reliability was calculated on the independent coded assessments. The consensus values were used in further analyses. This method, of creating a final, gold-standard code from consensus, is the standard in content analysis (Weber, 1990; Smith, 2000).

### 2.5. Analyses

The data consists of 1,875 Polish Twitter posts that were each coded by three annotators. Nine annotators were divided into three groups, which each coded a set of these tweets; there was no overlap in posts coded across the groups. Note that while a total of 1,875 posts were coded, model comparisons require models to be fit to the same data, thus certain analyses have fewer posts in the model to allow comparability across models due to missing data. Inter-annotator reliability for binary consensus CAT items was calculated *via* Fleiss’s Kappa and numeric consensus items for CAT and SMEmo were calculated *via* intraclass correlation coefficients [ICC(3,k)].

#### 2.5.1. RQ1

For the first research question, we explored the possible elements for characterizing cute stimuli and how those elements related to each other in the most parsimonious way. For the binary items indicating discrete cute content on the CAT measure, we calculated a sum score, representing the total number of cute attributes present in a post. We investigated how well this scale correlated with the six CAT items ranging 0–100 indicating continuous cute schemas, as well as the SMEmo-Cute Content to examine the convergent validity of this scale. We used Spearman rank-order correlations to calculate all correlations.

We then examined whether the six CAT items rated on the 0–100 scale could be combined into one unidimensional scale. To this end, we conducted an exploratory factor analysis (EFA) using principal axis factoring (PAF). PAF was conducted in R version 4.1.3 (R Core Team, 2022) using the psych package, version 2.1.9 (Revelle, 2021). To determine the number of factors, we examined a scree plot, the results of a parallel analysis (Horn, 1965), and the eigenvalues for each factor to determine the maximum number of factors that could feasibly be extracted. For PAF models with multiple factors, we used an oblimin rotation. Then, we fit PAF models containing one factor up to the maximum number and examined how the number of factors impacted interpretability of the factor, total variation among the items explained by the factors, and the internal consistency of the resulting scales. These results determined whether and in what way these items could be combined into a scale.

#### 2.5.2. RQ2

In the second research question, our goal was to explore the relationships between characteristics of cute content as measured by CAT (both discrete features, e.g., the presence of specific cute content, and continuous features, e.g., the magnitude of cute attributes); and the experience of cute emotional reaction (measured by the level of kama muta emotion evoked by that content as measured by the KAMMUS Two subscales and the level of heartwarming feeling as measured by the HSM scale). Specifically, we were interested in the extent to which the items that comprise CAT can predict both the KAMMUS Two subscales and the HSM (above and beyond the KAMMUS Two), as this would suggest that cute social media posts predict the extent to which individuals experience cute emotional reactions. Zickfeld et al. (2019) suggest examining the relationships between each of the KAMMUS subscales rather than combining them in some way. We expected some moderate level of relationship between characteristics of cute content (CAT) and emotional reactions evoked by cute content (KAMMUS Two and HSM), but would not expect there to be a tight relationship as one aspires to be a more objective content measure while the other reflects human emotions overtly; indeed, other variables outside of scope here may affect the strength of emotional reactions (e.g., various aspects of personal history and demographics).

We fit a zero-intercept mixed-effects hurdle model in R using the glmmTMB package, version 1.1.4 (Brooks et al., 2017). A hurdle model is a type of mixture model (Mullahy, 1986) with two parts: a binomial logistic regression and a regression with a conditional outcome distribution with only positive support (such as a truncated Poisson distribution or gamma distribution). Hurdle modeling was selected to account for the large amount of zeros present in the subscale values as expected for social media data, allowing the model to jointly estimate two models: one predicting whether or not the subscale values are 0 (binomial logistic mixed-effects model), and one predicting–when the subscale values are >0–the magnitude of the subscale values (in our case, a gamma mixed-effects model). This exploratory model regressed KAMMUS Two values on six binary dummy variables (one for each of the original KAMMUS Two subscales), and the two-way interaction term between each of the KAMMUS Two subscales and a single CAT term. This parameterization, which excludes the intercept and CAT main effect, results in an estimate of the relationship between the CAT term included in the model and each of the KAMMUS Two subscales. Each model was fit with cross-classified random intercepts for reviewer and post URL.

We determined which model (and thus CAT term) best predicted KAMMUS Two subscale values by comparing each non-nested model’s AIC. When the best model was selected, we then determined the maximal feasible random effects structure to include from the results of likelihood ratio tests (LRT) testing whether the variance and covariances of the random slopes were zero. LRTs were conducted for each possible random slope until the model failed to converge. In addition to finding the model that best predicted the validated KAMMUS Two values, we also fit models using the same procedure that included the HSM items created for this study. All models were fit using a gamma distribution to account for the continuous nature of the mean values as well as the right-skewed nature of the values. To ensure comparability across models, all cases with missing values were removed, resulting in 1,722 posts for analysis.

#### 2.5.3. RQ3

For the third research question, we examined how well both the KAMMUS Two subscales, the HSM scale, and the CAT items predicted the group-level gestalt consensus value on the SMEmo-Cute Content scale. First, we conducted a model selection process regressing SMEmo-Cute Content value on CAT items only, using LRTs on nested models to determine which CAT items best predict SMEmo-Cute Content value. These models included random intercepts by annotation group. Once the optimal fixed effects structure was determined, we used a similar procedure to determine which terms should have random slopes. The same process was used for predicting SMEmo-Cute Content value with the KAMMUS Two subscales and then again to add the HSM scale, though random slopes at the individual annotator level were also considered in these models. This modeling procedure also used a hurdle model as in RQ2, again using a gamma distribution to account for the skewed nature of the SMEmo-Cute Content value.

## 3. Results

**Figure 1:**
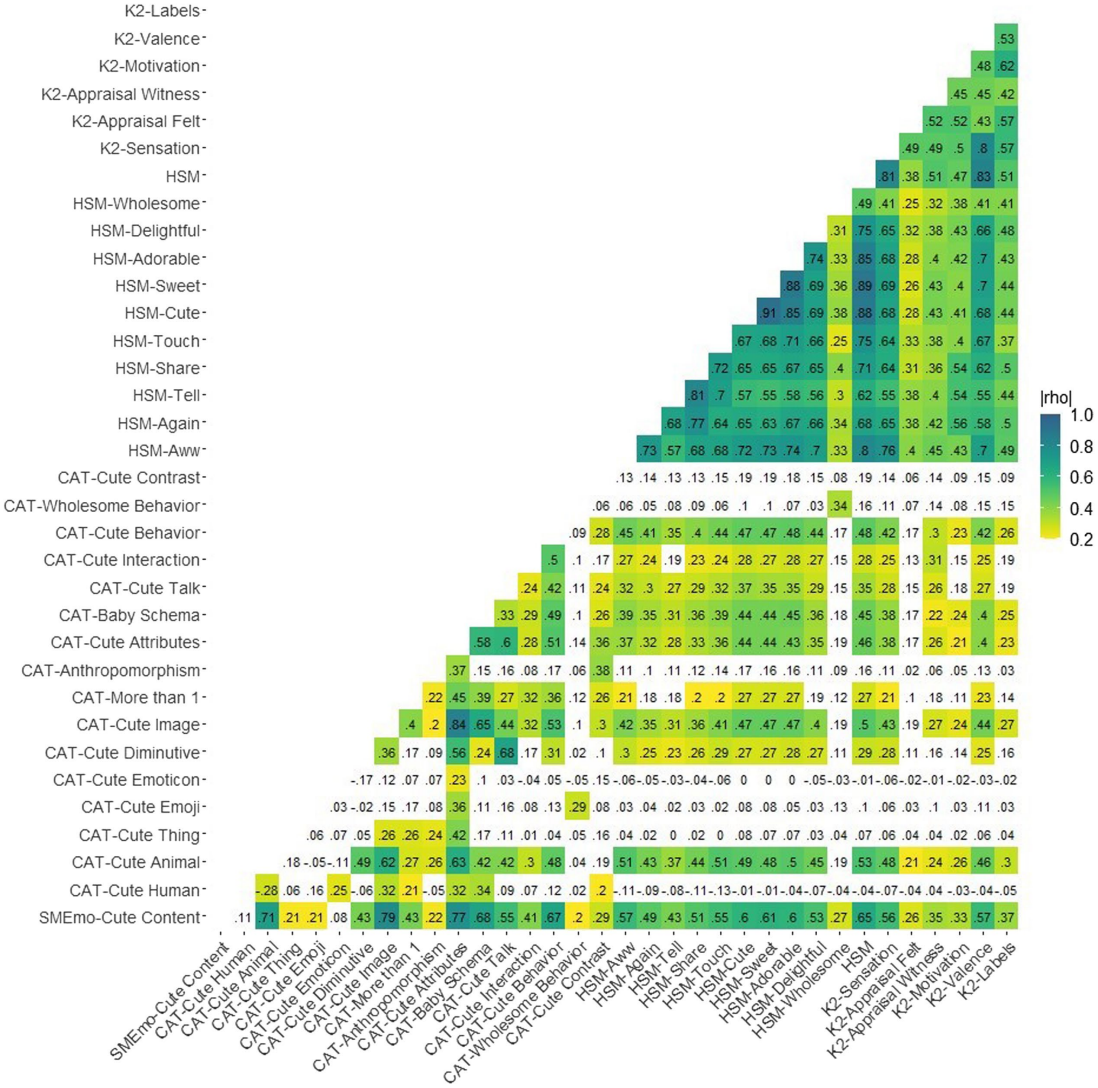
Spearman rho correlation matrix for SMEmo-Cute Content, CAT, HSH, and KAMMUS Two subscales.

Table 1 shows the descriptive statistics and interrater reliability for each consensus value and rating in the data. All interrater reliabilities for consensus values were acceptable above 0.70 with the exception of CAT-Anthropomorphism at 0.44. Notably, most of these measures are heavily zero-inflated. Figure 1 shows the Spearman rank-order correlations among the SMEmo-Cute Content consensus value, CAT item consensus values, the scale of binary CAT items describing total number of cute attributes, KAMMUS Two subscales, and the HSM scale. Overall, the KAMMUS Two (K2) subscales and the HSM scale are moderately to strongly correlated, and the HSM is most highly correlated with, in order, K2-Valence, K2-Sensation, and SMEmo-Cute Content. The CAT items are typically weakly intercorrelated with each other, with the exception of some of the binary CAT items like CAT-Cute Animal. The SMEmo-Cute Content consensus value tends to be weakly to moderately correlated with all of the items and subscales, with particularly strong correlations for, in order, CAT-Cute Image, CAT-Discrete Attributes, and CAT-Cute Animal. The highest correlations for SMEmo-Cute Content and the K2 are for K2-Valence and K2-Sensation, but it is more highly correlated with the HSM than any K2 subscale.

**Table 1:** Descriptive statistics of CAT and SMEmo-Cute consensus values and KAMMUS Two and HSM ratings.

**Binary consensus items (CAT)**					
Variable	Number of posts	Mean	SD	Reliability	
CAT-Cute human	1,837	0.15	0.36	0.75	
CAT-Cute animal	1,838	0.42	0.49	0.87	
CAT-Cute thing	1,838	0.11	0.32	0.73	
CAT-Cute emoji	1,838	0.22	0.41	0.82	
CAT-Cute emoticon	1,841	0.08	0.28	0.78	
CAT-Cute diminutive	1,837	0.45	0.50	0.77	
CAT-Cute image	1,840	0.53	0.50	0.81	
CAT-anthropomorphism	1,611	0.07	0.25	0.44	
**Numeric consensus items (SMEmo and CAT)**					
Variable	Number of posts	Mean	SD	Median	Reliability
SMEmo-Cute content	1,838	25.57	30.12	10	0.95
CAT-Baby schema	1,834	12.9	24.54	0	0.91
CAT-Cute talk	1,832	6.83	10.69	5	0.70
CAT-Cute interaction	1,840	4.80	15.43	0	0.87
CAT-Cute behavior	1,835	9.73	20.53	0	0.87
CAT-Wholesome behavior	1,794	4.20	12.21	0	0.78
CAT-Cute contrast	1,804	1.64	7.25	0	0.73
CAT-Discrete attributes	1,875	1.99	1.61	2	-
**Numeric rating items (KAMMUS Two and HSM)**					
Variable	Number of posts	Mean	SD	Median	
K2-Sensation	1,856	0.21	0.42	0	
K2-Appraisal felt	1,856	0.14	0.59	0	
K2-Appraisal witness	1,856	0.39	1.04	0	
K2-Motivation	1,856	0.19	0.67	0	
K2-Valence	1,856	1.22	1.86	0	
K2-Labels	1,856	0.25	0.81	0	
HSM	1,856	0.72	1.29	0	

Reliability for numeric items (i.e., on 0–100) scale is calculated with ICC(3,k). Reliability for binary items is calculated with Fleiss’s Kappa. Reliability is reported as the weighted average reliability score across the three groups, weighted by the number of posts rated in that group.

*RQ1*: What is the most parsimonious way to characterize the relationships between possible attributes of cute social media content in the proposed Cuteness Attributes Taxonomy (CAT)?

We conducted a PAF, specifically focusing on the consensus values of the six continuous items rated on a 0–100 scale in the CAT. A parallel analysis suggested two factors should be extracted, though a scree plot of the data showed poor support for factors beyond the first. Thus, we fit for both a one-and two-factor model. Ultimately, neither model was particularly strong: the one-factor model explained 30% of the variability in the items, while including the second factor only increased this to 35%. Additionally, the second factor in the two-factor model had only one item with a loading over 0.30.

Thus, the one-factor model was preferable due to the second factor consisting of only a single item. The results of this model are shown in Table 2. However, the one-factor model had low internal consistency ( α=0.67 ). Even when the two items that loaded the worst onto the scale are excluded (CAT-Wholesome Behavior and CAT-Cute Contrast), the reliability increased marginally ( α=0.70 ) although technically acceptable at a 0.70 threshold. Additionally, the consensus values on all of the CAT items ranged from 0 to 100 (with the exception of CAT-Cute Contrast, which ranged from zero to 93.33), indicating that the full scale was used for each item to some extent to describe posts with cute content. Based on the combination of acceptable interrater reliability for individual numeric consensus items on the CAT and limited evidence (marginally acceptable reliability and low variance explained) from EFA that the numeric items are unidimensional, we concluded that the CAT numeric consensus items were best treated individually, rather than as a scale, and further analyses that incorporate these scaled CAT items use them individually.

**Table 2:** Results of principal axis factoring of CAT values.

Item	Loading
CAT-Baby schema	0.56
CAT-Cute talk	0.42
CAT-Cute interaction	0.61
CAT-Cute behavior	0.91
CAT-Wholesome behavior	0.25
CAT-Cute contrast	0.29
Variance explained	0.30
Cronbach’s α	0.67

Because the eight binary items on the CAT simply identify whether or not a post contained a certain discrete element, we chose to create a sum score (CAT-Discrete Attributes) of these items that quantify the total number of cute attributes present in a post. Overall, these items tended to be weakly intercorrelated at best, though the presence of either an animal or diminutive language in a post was moderately positively correlated with the presence of an image. Thus, the sum value is a reasonable way to create a summary measure of several discrete cute attributes a post may have. The total number of cute attributes in a post was positively correlated with all of the CAT numeric consensus items rated on a 0–100 scale, though the correlation with wholesome behavior was low (0.14). Refer back to Figure 1 for an overall correlation matrix to see the CAT item correlations.

*RQ2*: Do attributes of cute content (as measured by the CAT) predict experiences of ‘cute’ emotional reaction, as measured by both the established metric KAMMUS Two and the proposed metric Heartwarming Social Media (HSM)? Does the HSM provide added value alongside the KAMMUS Two in this context?

Based on the results of RQ1, we chose to examine how well the number of discrete cute attributes (CAT-Discrete Attributes) in a post and the value on each of the individual CAT scaled items predicted an individual’s kama muta reaction from a post as measured by the previously validated KAMMUS Two (K2) subscales and the HSM scale. We were specifically interested in which pieces of the CAT (as measures of cute-related content) would best predict K2 and HSM values (as indices of cute-related emotional responses).

To assess how well each CAT item predicted the K2 subscales and HSM scale, seven zero-intercept generalized mixed effects hurdle models were fit, each with a categorical indicator for each KAMMUS Two subscale and the HSM plus their two-way interactions with one of the seven CAT variables (the six scaled consensus items and the CAT-Discrete Attributes score) at a time as the predictors and the K2/HSM scale values as the outcomes. To ease interpretation, the model coefficients have been exponentiated. For the zero-inflated model, this results in an odds ratio (OR), the multiplicative increase in the odds of the post having a value of zero (not being present); for the conditional model, the resulting incidence rate ratio (IRR) is the expected multiplicative increase in the non-zero outcome value for a unit increase in the predictor.

Table 3 shows the relative strength of each CAT item as a predictor of KAMMUS Two subscales and HSM using AIC. Regardless of whether the HSM items are included, the most predictive CAT items are the same: the total number of cute attributes is the best predictor of the KAMMUS Two subscales values, followed by the cute behavior and baby schema values. While the number of cute attributes was the strongest predictor, all of the CAT items generally predicted higher values on the KAMMUS Two subscales, though the weaker predictors had some non-significant associations.

**Table 3:** AIC values for models predicting KAMMUS Two and HSM values.

Predictor	K2 only	K2 and HSM
CAT-Discrete attributes	36,329	46,325
CAT-Cute behavior	36,510	46,643
CAT-Baby schema	36,617	46,664
CAT-Cute talk	36,929	47,209
CAT-Cute interaction	36,869	47,225
CAT-Wholesome behavior	37,024	47,397
CAT-Cute contrast	37,138	47,487

Smaller values reflect better model fit for a column (the values in the K2 only column are not directly comparable to the K2 and HSM set of models).

Because the total number of cute attributes was the strongest predictor, it was used to fit a final model to predict the results for the model using it as a predictor of KAMMUS Two subscale values (Table 4) and KAMMUS Two subscale values including the HSM scale (Table 5). These models did not need to exclude posts with missing data on any CAT scale, thus the number of posts in these final models is 1,854. These final models also considered potential random slopes, with the maximal feasible model containing a random slope for outcome scale type by Annotator. Regardless of whether the HSM scale is included, the total number of cute attributes is positively associated with all K2 subscale values, which suggests that the presence of cute content predicts the viewer experiencing kama muta.

**Table 4:** K2:CAT-discrete attributes model.

Model: SubscaleRating ~ 0 + Subscale + Subscale:CuteAttributes + (Subscale|Annotator) + (1|URL)				
Model dispersion parameter: 0.28				
**Fixed effects**				
Fixed effect	Estimate	Incidence rate ratio	Std. error	p-value
**Conditional model**				
K2-Appraisal felt	−0.40	0.67	0.21	0.061
K2-Appraisal witness	−0.09	0.91	0.21	0.655
K2-Labels	−0.02	0.98	0.14	0.893
K2-Motivation	−0.46	0.63	0.22	0.037
K2-Sensation	−1.18	0.31	0.16	<0.001
K2-Valence	0.80	2.22	0.09	<0.001
CAT-Discrete attributes:K2-Appraisal felt association	0.11	1.11	0.02	<0.001
CAT-Discrete attributes:K2-Appraisal witness association	0.11	1.12	0.02	<0.001
CAT-Discrete attributes:K2-Labels association	0.07	1.07	0.02	0.001
CAT-Discrete attributes:K2-Motivation association	0.10	1.10	0.02	<0.001
CAT-Discrete attributes:K2-Sensation association	0.14	1.15	0.01	<0.001
CAT-Discrete attributes:K2-Valence association	0.09	1.10	0.01	<0.001

Zero-inflation model				
Fixed effect	Estimate	Odds ratio	Std. error	*p*-value
K2-Appraisal felt	8.19	3610.72	1.56	<0.001
K2-Appraisal witness	4.90	134.62	0.53	<0.001
K2-Labels	5.39	218.66	0.48	<0.001
K2-Motivation	6.93	1021.75	0.97	<0.001
K2-Sensation	3.52	33.97	0.34	<0.001
K2-Valence	3.35	28.43	0.33	<0.001
CAT-Discrete attributes:K2-appraisal Felt association	−0.91	0.40	0.06	<0.001
CAT-Discrete attributes:K2-Appraisal witness association	−0.88	0.42	0.05	<0.001
CAT-Discrete attributes:K2-Labels association	−0.93	0.39	0.05	<0.001
CAT-Discrete attributes:K2-Motivation association	−1.00	0.37	0.06	<0.001
CAT-Discrete attributes:K2-Sensation association	−1.07	0.34	0.04	<0.001
CAT-Discrete attributes:K2-Valence association	−1.11	0.33	0.04	<0.001

Random effect	Variance	Std. deviation	Correlation				
			Intercept	Appraisal witness	Labels	Motivation	Sensation
**Conditional model**							
*Annotator:*							
Intercept	0.34	0.58					
Appraisal witness	0.20	0.45	−0.34				
Labels	0.12	0.34	−0.82	−0.10			
Motivation	0.06	0.24	−0.10	0.03	0.29		
Sensation	0.29	0.54	−0.66	0.28	0.73	0.11	
Valence	0.13	0.36	−0.97	0.26	0.74	−0.1	0.55
*URL:*							
Intercept	0.16	0.40					
**Zero-inflation model**							
*Annotator*:							
Intercept	20.40	4.52					
Appraisal Witness	17.58	4.19	−0.94				
Labels	12.16	3.49	−0.98	0.92			
Motivation	7.14	2.67	−0.82	0.70	0.84		
Sensation	17.39	4.17	−0.98	0.90	0.91	0.79	
Valence	18.12	4.26	−0.98	0.90	0.92	0.83	1.00
*URL:*							
Intercept	3.90	1.98					

**Table 5:** K2 + HSM:CAT-Discrete attributes model (with HSM subscale).

*Model: SubscaleRating ~ 0 + Subscale + Subscale:CuteAttributes + (Subscale|Annotator) + (1|URL)*								
Model dispersion parameter: 0.33								
**Fixed effects**								
Fixed effect	Estimate	Incident rate ratio	Std. error		*p*-value			
**Conditional model**								
K2-Appraisal felt	−0.61	0.55	0.21		0.004			
K2-Appraisal witness	−0.23	0.80	0.21		0.280			
HSM	−0.61	0.54	0.08		<0.001			
K2-Labels	−0.23	0.80	0.14		0.105			
K2-Motivation	−0.65	0.52	0.22		0.003			
K2-Sensation	−1.36	0.26	0.16		<0.001			
K2-Valence	0.67	1.96	0.10		<0.001			
CAT-Discrete attributes:K2-Appraisal felt association	0.15	1.16	0.03		<0.001			
CAT-Discrete attributes:K2-Appraisal witness association	0.14	1.15	0.02		<0.001			
CAT-Discrete attributes:HSM	0.28	1.33	0.02		<0.001			
CAT-Discrete attributes:K2-Labels association	0.10	1.11	0.02		<0.001			
CAT-Discrete attributes:K2-Motivation association	0.13	1.14	0.03		<0.001			
CAT-Discrete attributes:K2-Sensation association	0.16	1.18	0.02		<0.001			
CAT-Discrete attributes:K2-Valence association	0.11	1.11	0.02		<0.001			
**Zero-inflation model**								
Fixed effect	Estimate	Odds ratio	Std. error		*p*-value			
K2-Appraisal felt	8.12	3376.65	1.36		<0.001			
K2-Appraisal witness	5.18	178.16	0.54		<0.001			
HSM	3.48	32.55	0.44		<0.001			
K2-Labels	5.70	297.56	0.48		<0.001			
K2-Motivation	7.27	1440.56	0.99		<0.001			
K2-Sensation	3.78	43.66	0.35		<0.001			
K2-Valence	3.58	36.05	0.34		<0.001			
CAT-Discrete attributes:K2-Appraisal Felt association	−0.98	0.38	0.06		<0.001			
CAT-Discrete attributes:K2---Appraisal Witness association	−0.94	0.39	0.05		<0.001			
CAT-Discrete attributes:HSM	−1.28	0.28	0.05		<0.001			
CAT-Discrete attributes:K2-Labels association	−1.00	0.37	0.05		<0.001			
CAT-Discrete attributes:K2-Motivation association	−1.06	0.35	0.06		<0.001			
CAT-Discrete attributes:K2-Sensation association	−1.14	0.32	0.05		<0.001			
CAT-Discrete attributes:K2-Valence association	−1.18	0.31	0.05		<0.001			
Random effect	Variance	Std. deviation	Correlation					
			Int.	App. Wit.	HSM	Labels	Mot.	Sens.
**Conditional model**								
*Annotator*:								
Intercept	0.30	0.55						
Appraisal witness	0.17	0.41	−0.24					
HSM	0.19	0.44	−0.94	0.22				
Labels	0.10	0.32	−0.76	−0.34	0.72			
Motivation	0.06	0.24	−0.12	0.06	0.33	0.10		
Sensation	0.22	0.46	−0.58	0.03	0.49	0.67	−0.05	
Valence	0.12	0.34	−0.91	0.10	0.77	0.72	−0.16	0.40
*URL:*								
Intercept	0.27	0.52						
**Zero-inflation model**								
*Annotator*:								
Intercept	12.46	3.53						
Appraisal witness	10.44	3.23	−0.90					
HSM	14.28	3.84	−0.95	0.89				
Labels	6.19	2.49	−0.96	0.84	0.85			
Motivation	4.06	2.01	−0.60	0.39	0.54	0.63		
Sensation	10.95	3.31	−0.96	0.83	0.97	0.85	0.58	
Valence	11.52	3.39	−0.96	0.82	0.95	0.86	0.66	0.99
*URL:*								
Intercept	4.53	2.13						

Additionally, when the HSM scale was included in addition to the KAMMUS subscales, the number of cute attributes was strongly associated with the value on this scale (IRR = 1.33, *p* < 0.001), more so than the K2 subscale (IRRs = 1.11–1.18, all *p*s < 0.001). The HSM scale showed strong reliability ( α=0.92 ), and a scree plot suggests that a one-factor PAF model is reasonable (see PAF modeling procedure for RQ1). This one factor explains 57% of the variability in the items and all of the items load onto the factor (Table 6).

**Table 6:** Results of principal axis factoring of HSM values.

Item	Loading
Said “Aww”	0.75
Wanted to have this experience again	0.57
Wanted to tell someone	0.57
Wanted to share this	0.68
Wanted to touch thing in post	0.80
It was cute	0.92
It was sweet	0.91
It was adorable	0.93
It was delightful	0.83
It was wholesome	0.45
Variance explained	0.57
Cronbach’s α	0.92

*RQ3*: Do experiences of attributes of cute content (CAT) predict gestalt cute social media content (indexed by SMEmo-Cute Content)? In the relationship between cute emotional reactions and cute social media content, does the newly developed Heartwarming Social Media (HSM) scale contribute above and beyond the established kama muta emotions (KAMMUS Two)?

To assess how well CAT, KAMMUS Two, and HSM predict the SMEmo-Cute Content value, we followed a model-building process that considered CAT and KAMMUS Two/HSM separately. As in RQ2, the models fit were mixed effects hurdle models with a gamma distribution. Because SMEmo-Cute Content is a consensus value and there were three annotator groups that judged non-overlapping posts, we included random intercepts by annotator group. Additionally, models including KAMMUS Two and HSM also included random slopes for each individual annotator. To build the individual CAT and KAMMUS Two/HSM models, we determined which CAT items to include as predictors from the results of LRTs as different items were added. Once we determined which fixed effects to include, we determined the maximal feasible random slope structure.

Table 7 shows the results for the CAT model. These models used 1,722 posts that contained no missing data among all considered predictors. All CAT items were included in the final model besides cute contrast; no random slopes were included in the final model because they did not improve model fit. In the zero-inflation model predicting whether the SMEmo-Cute Content value is zero or non-zero, only the CAT-Discrete Attributes metric (the total number of cute attributes), the CAT-Wholesome Behavior scale, and CAT-Cute Talk scale are significant, with all three associated with non-zero values when all other CAT metrics are held constant. In other words, as these measures increased, so too did the probability that cute content would be present as measured by SMEmo-Cute Content. However, in the conditional model predicting the intensity of posts with non-zero SMEmo-Cute Content, *all* CAT metrics predict higher content values when holding each other constant. This finding suggests that the SMEmo-Cute Content measure, which is an overall, holistic value, may implicitly include elements described by the CAT items.

**Table 7:** Model predicting SMEmo-Cute content with CAT items.

*Model: CuteContent ~ BabySchema* + *CuteTalk* + *CuteInteract* + *WholesomeBehav* + *CuteBehav* + *CuteAttributes* + *(1|Group)*				
Model dispersion parameter: 0.44				
**Conditional model**				
Fixed effects	Estimate	Incidence rate ratio	Std. error	*p*-value
Intercept	2.12	8.36	0.06	<0.001
CAT-Baby schema	0.01	1.01	0.001	<0.001
CAT-Cute talk	0.004	1.00	0.002	0.021
CAT-Cute interactions	0.004	1.00	0.001	0.003
CAT-Wholesome behavior	0.004	1.00	0.001	0.004
CAT-Cute behavior	0.01	1.01	0.001	<0.001
CAT-Discrete attributes	0.29	1.34	0.02	<0.001
Random effects	Variance	Std. deviation		
Group intercept	<0.00001	0.00001		
**Zero-inflation model**				
Fixed effects	Estimate	Odds ratio	Std. error	*p*-value
Intercept	2.72	15.24	0.17	<0.001
CAT-Baby schema	−0.07	0.93	0.04	0.055
CAT-Cute talk	−0.05	0.95	0.02	0.011
CAT-Cute interactions	−0.06	0.95	0.08	0.501
CAT-Wholesome behavior	−0.11	0.90	0.02	<0.001
CAT-Cute behavior	−0.38	0.68	0.20	0.055
CAT-Discrete attributes	−2.18	0.11	0.16	<0.001
Random effects	Variance	Std. deviation		
Group intercept	<0.00001	0.0001		

Table 8 shows the results for the KAMMUS Two model including the HSM scale. These models used 1,835 posts with no missing data across all of the measures. Every KAMMUS Two subscale was retained in the final model; although the LRT for including motivation was non-significant (*p* = 0.063), we chose to retain it to prevent excluding only one of the previously validated KAMMUS Two subscales. Random slopes of HSM and motivation by individual annotator were included. A model was also tested excluding the HSM scale, but model fit was significantly worse and thus is not presented. Holding all other subscales constant, K2-Valence and HSM predicted both non-zero SMEmo-Cute Content values (valence OR = 0.77, *p* < 0.001; HSM scale OR = 0.03, *p* < 0.001) and higher SMEmo-Cute Content values (valence IRR = 1.06, *p* < 0.001; HSM scale IRR = 1.29, *p* < 0.001) for posts with non-zero values. Additionally, the K2-Appraisal Witness subscale predicted non-zero SMEmo-Cute Content (OR = 0.76, *p* = 0.002), and the K2-Sensation subscale predicted higher values for posts with non-zero values (IRR = 1.17, *p* = 0.004). However, two subscales were not always associated with higher SMEmo-Cute Content values: conditional on all other subscales, the K2-Appraisal Felt subscale was associated with posts having a SMEmo-Cute Content value of zero (OR = 1.44, *p* = 0.002), and the K2-Labels subscale was associated with lower SMEmo-Cute Content values among posts with non-zero cute content (IRR = 0.93, *p* = 0.002). This finding may suggest that certain elements of kama muta are not felt when engaging with cute social media content.

**Table 8:** Model predicting SMEmo-Cute content with KAMMUS Two and HSM scales.

*Model: CuteContent ~ Sensation* + *AppraiseFelt* + *AppraiseWitness* + *Motivation* + *Valence* + *Labels* + *HSM* + *(1|Group)* + *(0* + *HSM* + *Motivation|Group: Annotator)*				
Model dispersion parameter: 0.57				
**Conditional model**				
Fixed effects	Estimate	Incident rate ratio	SE	*p*-value
Intercept	3.13	22.78	0.11	<0.001
K2-Sensation	0.16	1.17	0.05	0.004
K2-Appraisal felt	0.004	1.00	0.03	0.899
K2-Appraisal witness	−0.01	0.99	0.01	0.644
K2-Motivation	−0.03	0.97	0.07	0.627
K2-Valence	0.05	1.06	0.01	<0.001
K2-Labels	−0.07	0.93	0.02	0.002
HSM	0.26	1.29	0.03	<0.001
Random effects	Variance	SD	Correlation	
Group: intercept	0.04	0.19		
Reviewer: HSM slope	0.01	0.08		
Reviewer: Motivation slope	0.01	0.10	−0.80	
**Zero-inflation model**				
Fixed effects	Estimate	Odds ratio	SE	*p*-value
Intercept	0.03	1.03	0.11	0.813
K2-Sensation	0.03	1.03	0.21	0.887
K2-Appraisal felt	0.37	1.44	0.12	0.002
K2-Appraisal witness	−0.27	0.76	0.09	0.002
K2-Motivation	0.57	1.77	1.24	0.644
K2-Valence	−0.27	0.77	0.05	<0.001
K2-Labels	0.09	1.10	0.12	0.459
HSM	−3.47	0.03	0.88	<0.001
Random effects	Variance	SD	Correlation	
Group: Intercept	0.03	0.18		
Annotator: HSM slope	6.07	2.46		
Annotator: Motivation slope	4.32	2.08	−0.76	

## 4. Discussion

The current study designed, tested, and provided initial validity evidence for new measures of cute social media content (Cuteness Attributes Taxonomy; CAT) and emotional reactions (Heartwarming Social Media; HSM), and tested whether kama muta, as measured by KAMMUS Two, is the emotional reaction evoked when being exposed to cute stimuli on social media and tested whether gestalt cute content (SMEmo-Cute Content) reflects what is measured by the CAT and is related to the HSM. Overall, our results show promising initial justification for both the CAT for the purpose of categorizing and quantifying the content of a “cute” post and the HSM measure for capturing cute emotional reaction, and that the HSM captures cute emotional reaction to cute social media content above and beyond the established KAMMUS Two.

### 4.1. Categorizing cute social media content

The most widely used approach to categorizing cute content relies on the theory of Kindchenschema, coined by Lorenz (1943), which focuses on physical attributes commonly associated with infant or young children (e.g., large head size, large eyes) and suggests these features evoke adult caretaking behaviors. Later approaches expanded beyond Kindchenschema to include non-baby schema characteristics that evoke positive emotions and approach motivation. We propose that in the social media context, baby schema is a necessary but not sufficient component of cuteness and how people react to it. We posit that, perhaps because of the nature of the context, cute social media content evokes approach motivations shown through sharing and other prosocial online behaviors, either in addition to or in place of evoking parental instincts. To that end, we developed and found initial validity evidence for CAT to include this expanded taxonomy of cuteness attributes for research in social media contexts.

As our results show, the Cuteness Attributes Taxonomy (CAT) measure predicts kama muta (as measured by KAMMUS Two), and cute emotional reaction (as measured by the proposed HSM). We tested several possible relationships between individual cute attributes within the CAT and found that the best conceptualization in this dataset was to treat each continuous cute attribute as independent measures of cuteness intensity (due to a lack of strong unidimensionality) and the discrete cute attributes as a sum score. In other words, the continuous cute attributes would be measures of how much each different cute attribute (or perhaps, another way to frame them would be as different schemas for cuteness) exists in the post, and the discrete attributes as an aggregate would be a measure of how much “cute” content a post contains. The discrete attributes help to make sense in a social media context where posts may contain multiple aspects of cute content that, if presented alone, may constitute “cute,” but together enhance or increase the amount of “cuteness” in a post. For example, a post containing an image of a pet dog (CAT item—cute animal and cute image) may be considered cute on its own, but the addition of a bow tie on the dog (CAT item—cute thing) and/or a cute caption (CAT item—cute emoji or cute diminutive) may increase the emotional reaction due to the additional cuteness attributes. The gestalt measure of cute content, SMEmo-Cute Content, which is an overall measure of the amount of “cuteness” in a post, was also predicted by both the individual continuous items of the CAT, and by the discrete item sum value, thus suggesting that the elements of the CAT are considered in the overall measure of cute content in a post.

Further, the sum score of CAT cute attributes was the best predictor of KAMMUS Two values, followed by the individual numeric items of cute behavior and baby schema values—but all CAT items generally predicted higher values on KAMMUS Two and on the HSM scale. Thus, the CAT measure captures characteristics of social media posts likely to evoke the kama muta emotion and heartwarming feelings on social media.

As in any research technique involving human annotation, using CAT is relatively time-consuming. However, the return on investment can be vast as granular information is collected on both the discrete features of cuteness and the degree to which different features are present in the post. Understanding how information and interactions online evoke responses can benefit from capturing the full range of the content features present. What is considered cute to one person might not be cute to another, might evoke a mixed reaction, or might vary within different contexts, mediums, or settings to which the CAT could be applied. As an example, consider a context in which critical information must be conveyed to the public, such as a public health emergency. Prior to releasing a social media campaign designed to inform and modify public behavior (e.g., to encourage hygienic practices), messages using different CAT attributes could be pilot tested to determine the likely reactions to the message, such as intent to engage in the target behaviors.

### 4.2. Emotional reaction to cute content

As theories of what constitutes cute content have expanded to include both the Kindcheschema and non-baby schema characteristics, the theories of cute emotion(s) have expanded to a view that includes cuteness as a stimulus to social behaviors and communal sharing (kama muta). As kama muta is an emotional experience largely studied offline, we posited that online experiences of “cute emotions” might encompass a different scope of reactions. To that end, we developed and provided initial validity evidence for the Heartwarming Social Media (HSM) scale. Generally, cute content evokes a positive, prosocial emotional reaction (e.g., heartwarming, moved, wholesome). The SMEmo-Cute Content is a gestalt measure of the amount of “cuteness” in a post. This holistic measure should be strongly related to the CAT measure (conceptually, both content measures) and also related, but to a lesser degree, to the KAMMUS Two subscales and HSM scale (conceptually relating a content measure to two emotional reaction measures). In other words, the more cute content in a post, the higher the experience of the cute emotions one should feel.

When examining the relationship between KAMMUS Two and HSM as predictors of SMEmo-Cute Content, including the HSM scale in addition to the KAMMUS Two improved model fit, indicating that in our dataset the HSM scale is an important predictor of a reaction to cute content on social media. Further, the HSM scale had a strong predictive relationship to the SMEmo-Cute Content value in every model; in fact it had the largest effect sizes in the model (KAMMUS Two and HSM predicting SMEmo-Cute Content), the strongest predictor of both the presence of gestalt cute content and also the strongest predictor of the magnitude (or intensity) of gestalt cute content. Conversely, not all the KAMMUS Two subscales were predictive in the expected direction. In the model without CAT, the Appraisal Felt subscale was predictive of a SMEmo-Cute value of zero (the absence of cute content), while the Labels subscale was predictive of lower SMEmo-Cute Content values. This result suggests that experiences of cute emotions may be expressed, experienced, or serve different purposes in the online environment vs. offline.

Interaction rituals, defined as periods of shared emotion and attention usually within a shared physical space and time, are typical of community membership and bonding and can take place in online mediated environments (Törnberg and Törnberg, 2022). Because the online environment lacks the synchrony of space and time, community is built primarily around the exchange of messages around a shared topic and less on physical cues, resulting in different intensity or requiring different responses from members to build community (DiMaggio et al., 2019; Törnberg and Törnberg, 2022). Similarly, there may be a difference between in-person and online experiences that evoke kama muta, where some KAMMUS Two subscales may be more typical of in-person interactions or experiences. As most measures of emotion are developed in and for offline contexts, this finding highlights the importance of validating existing measures in new contexts and of developing online-specific measures where appropriate. We recommend researchers use these measures according to the goals of their future studies. For instance, the CAT would describe specific content features, whereas the HSM scale can measure the heartwarming feelings when exposed to cute social media.

### 4.3. Limitations

There are several limitations to note for this study. First, our annotators’ demographic characteristics skewed toward younger, female, and highly educated, which could potentially affect annotation of cute content. Second, all posts were pulled from Polish Twitter, which is not the predominant social media platform in Poland, where all the annotators lived. The use of Polish language and culture social media could affect the generalizability of these results to other cultures and languages. Future replication studies should focus on similar cultures and samples, such as Lithuanian in Lithuania, but also widely studied languages such as English in the United States, and more distant to Polish languages such as Mandarin Chinese in China. Replication studies should also utilize a broader range of platforms and annotators. Further research to validate these measures may benefit from gathering further convergent validity evidence as mentioned, and divergent validity evidence, examining whether the CAT and HSM are separable from decidedly non-cute emotions, such as anger or disgust, and whether they are partially separable from related but distinct emotions, such as love and happiness. Finally, the nature of the multi-step procedure for data collection and sampling we utilized might also affect the generalizability of these findings. Because it involved multiple decision points throughout the process (e.g., keywords we used) and chance (random selection of tweets), we acknowledge that any divergences in these decisions could possibly yield different outcomes. As a result, we did not collect a truly random sample of posts, and acknowledge that these posts may not be representative of most Polish Twitter posts. However, by selecting posts likely to have cute content, we were able to tease apart different aspects of cuteness without spending precious annotator time on huge quantities of irrelevant content.

### 4.4. Practical and theoretical implications

The current study contributed three new measures to use in the investigation of cute content and emotions on social media. Anecdotally, cute content on social media is described as widely experienced. There are Reddit threads where users, mostly with humor, ask other Redditors what the purpose of the internet is, to which the top answer is always “cats.” Separately, entire accounts on various social media platforms are created and dedicated to sharing cute content (e.g., Reddit r/aww, Twitter’s We Rate Dogs). The prevalence and wide range of cute content, and the purposes to which such content is used, indicates it is worthy of study. Marketing and advertising often draw on cute content in their attempts to persuade (Nenkov and Scott, 2014), as do medical professionals in health messaging or public service announcements (Lien and Wu, 2021). There are also cases of terrorists using cute imagery (e.g., kittens) in recruitment or propaganda messaging (Farwell, 2014; Whitehead, 2016). These appeals to emotion (pathos) are considered a cornerstone of rhetoric (Aristotle, 2015, 350 B.C.E.), and communal emotional experiences are an important part of building shared, group identity (Cialdini, 2021). Both aspects are important components of persuasion used for good or ill (Cialdini, 2021). The ability to characterize, quantify, and predict the prevalence of cute content, its interaction with other topics and persuasive messages, and its propagation through social media and theoretically corresponding offline effects, is useful both for future theory and application.

Traditional explanations of cute emotions posit that they evoke a caretaking response based on the cute characteristics that resemble human infant characteristics, and that this response has generalized across other species to explain why we find baby animals cute. However, emotional reactions to cute content are complex and multifaceted, and do not always suggest a caretaking response. For instance, cute aggression is an example of a multifaceted response to the “cute emotion” (Stavropoulos and Alba, 2018). This reaction may be seen in response to many cute posts on social media, where responses to the cute content often veer into hyperbole in describing one’s reaction to cute things (e.g., “It’s so fluffy, I’m going to die!,” a meme from the movie *Despicable Me*). This reaction suggests aggression rather than caretaking, though generally, no aggressive intent is actually present.

We theorize that cute emotional responses may serve an alternative or complementary purpose to caretaking: to initiate and engage in social interaction or build social connection. Social media is a unique medium of communication that lacks certain social cues, such as body language. In the absence of those social cues, how might one indicate that they are open to interaction with others? Perhaps by sharing content which invites communal (positive) emotional experiences through cute posts. Research has also shown that people deliberately engage with cute social media content to regulate their mood (Myrick, 2015), and anecdotal evidence suggests some individuals request or share cute content with others to help regulate moods and provide social support. For example, some social media users will post requests for cute animal photos when having a bad day. Not only does this request usually elicit the requested content, but often results in social connection and interaction with others who wish the original poster well or offer to connect in other ways. Meanwhile, sharing cute pictures of children might connect one with other parents or caretakers, providing social support while raising or caring for children. Perhaps different kinds of cute content serve different, or multiple, purposes in social media?

Our findings also have implications for emotion and related theories. While kama muta is linked to communal sharing (Fiske et al., 2017), it is unlikely to be the only emotion indicative of that kind of relational structure. Future research can examine the pattern of emotions, such as love, which should also be related to communal sharing. The work on kama muta has been developing in parallel with semantic space theory, a greatly expanded discrete emotion approach (Cowen and Keltner, 2021). Research developing that theory has examined a variety of different emotions. In a study examining experiences from videos, cuteness was not called out specifically as a separate emotion, but the study included videos of baby and cute animals, which were rated with moderate levels of *adoration* (Cowen and Keltner, 2021).^13^ Our work suggests that cute reactions are not limited to adoration, and future research could continue to distinguish between related emotions.

## 5. Conclusion

While cute content is prevalent on social media, previous research has focused on cuteness characteristics and emotions it evokes in offline settings. The concept of cuteness is difficult to tackle in research studies as the perception of cuteness tends to be subjective and has largely focused on one perspective of cuteness attributes (baby schema). To this end, the current study presented and supported two new measures that can be used in quantifying an expanded set of the characteristics of cute social media content (CAT and HSM measures). This study also provided evidence that cute social media content evokes kama muta as measured by previously validated KAMMUS Two (Zickfeld et al., 2019); however, not all subscales of KAMMUS were equally sensitive to cuteness in this context. This argues for the necessity of measures of user reactions to cute stimuli specifically developed for social media contexts such as the HSM measure presented here. A greater understanding of the dimensions of this phenomena, and of how to measure it, is necessary and useful to support future research on the role of cute in social media sharing, production, and influence.

## Data availability statement

The raw data supporting the conclusions of this article will be made available by the authors, without undue reservation, in compliance with Twitter’s data use policy.

## Author contributions

SP and EG conceptualized the study. EG, SP, NP, and KJ designed the study. SP, CR, and EG acquired funding. CR and EG sampled data for investigation. EG led the investigation. PS, NP, CR, MJ, and EG curated data. PS performed statistical analyses. NP and MJ provided statistical expertise. EG, KJ, PS, SP, NP, and CR wrote the manuscript. All authors contributed to the article and approved the submitted version.

## Funding

This material is based upon work supported, in whole or in part, with funding from Minerva Research Initiative/Office of Naval Research grant #N00014-19-1-2506 and UMD ARLIS internal funds.

## Acknowledgments

We thank Cody Buntain for collecting data and for his suggestions in the early stages of this work and Petra Bradley for her contribution to developing the scales. We are grateful to Agata Bieniek, Anna Kostrzewa, Agata Kuzia, Klaudia Kuźnicka, Małgorzata Perczak-Partyka, Rafał Rosiak, Laura Russak, Ewa Szczepska, and Marta Urbańska-Łaba for data annotation and Anna Prince for assistance in sampling. We also thank Rebecca Goolsby for her enthusiastic support for this project, as well as two reviewers and the editor, whose feedback improved this article.

## Conflict of interest

The authors declare that the research was conducted in the absence of any commercial or financial relationships that could be construed as a potential conflict of interest.

## Publisher’s note

All claims expressed in this article are solely those of the authors and do not necessarily represent those of their affiliated organizations, or those of the publisher, the editors and the reviewers. Any product that may be evaluated in this article, or claim that may be made by its manufacturer, is not guaranteed or endorsed by the publisher.

## Author disclaimer

Any opinions, findings and conclusions or recommendations expressed in this material are those of the authors and do not necessarily reflect the views of the University of Maryland, College Park and/or any agency or entity of the US Government.

## Footnotes

^1^See Paletz, S. B. F., Johns, M., Murauskaite, E., Golonka, E., Pandža, N. B., Rytting, C. A., Buntain, C., and Ellis, D. Emotional content and sharing on Facebook: A theory cage match. Under review.

^2^For methodology and validation, see Paletz, S. B. F., Golonka, E., Pandža, N. B., Stanton, G., Ryan, D., Adams, N., Rytting, C. A., Murauskaite, E., Buntain, C., Johns, M., and Bradley, P. Social media emotions annotation guide (SMEmo): Development and initial validity. Under review.

^3^When used as a theoretical construct in the scientific literature in English, it does not have the erotic connotation it does in Sanskrit (http://kamamutalab.org/).

^4^We included all forms for the Nominative and Genitive cases for all genders and numbers.

^5^For a description of the selection criteria, see Paletz, S. B. F., Johns, M., Murauskaite, E., Golonka, E., Pandža, N. B., Rytting, C. A., Buntain, C., and Ellis, D. *Emotional content and sharing on Facebook: A theory cage match*. Manuscript under review.

^6^This new corpus used for Stages 2–4 does not use the same list of sociopolitical influencers as Stage 1, but is open to anyone using Twitter in Poland.

^7^For methodology and validation, see Paletz, S. B. F., Golonka, E., Pandža, N. B., Stanton, G., Ryan, D., Adams, N., Rytting, C. A., Murauskaite, E., Buntain, C., Johns, M., and Bradley, P. *Social media emotions annotation guide (SMEmo): Development and initial validity.* Under review.

^8^Annotators also assessed their own emotional reaction to cute content in the post on the same 0–100 scale (SMEmo-Cute Reaction, not used in this study).

^9^We used the Polish translation of KAMMUS Two from Zickfeld et al. (2019). Responses in the English version were: 0 means *not at all*, 6 means *a lot*.

^10^A similar item (I wanted to have this experience all over again with others) was in earlier versions of KAMMUS; while that question emphasized communal sharing, the current question emphasizes common social media behaviors of reading or viewing content multiple times.

^11^A similar item (I was eager to tell my friends or family about the experience) was in earlier versions of KAMMUS; the current question extends it to larger online communities.

^12^This item targets online engagement behaviors, e.g., retweeting, sharing, commenting.

^13^https://s3-us-west-1.amazonaws.com/emogifs/map.html#
