# Supplementary material for: The construct of cuteness: A validity study for measuring content and evoked emotions on social media
Source: Front Psychol. 2023 Mar 3;14:1068373. doi: 10.3389/fpsyg.2023.1068373 (PMC10020712; doi:10.3389/fpsyg.2023.1068373)
Supplement: Supplementary file 1 [file Table_1.pdf]

Supplementary Material

The Construct of Cuteness: A Validity Study for Measuring Content and Evoked Emotions on Social Media

Ewa M. Golonka[1*], Kelly M. Jones[1], Patrick Sheehan[2 †], Nick B. Pandža[1 †], Susannah B. F. Paletz[3], C. Anton Rytting[1], Michael A. Johns[4]

[1]Applied Research Laboratory for Intelligence and Security, University of Maryland, College Park, MD, United States

[2]Department of Human Development and Quantitative Methodology, University of Maryland, College Park, MD, United States

[3]College of Information Studies, University of Maryland, College Park, MD, United States

[4]Institute for Systems Research, University of Maryland, College Park, MD, United States

*** Correspondence:**
Ewa M. Golonka
egolonka@umd.edu

[†] These authors share third authorship

1 Social Media Emotions Annotation Guide - Cute Content (SMEmo-Cute Content)

The full and current version of Social Media Emotions Annotation Guide is available by request from the authors (see also https://emotionsinsocialmedia.umd.edu/). In the guide, annotators are directed how and what to code for each social media post in general, and then the guide offers over 20 distinct emotions and affective states (e.g., anger, which ranged from irritation to rage/wrath; shame/embarrassment/guilt; happiness/joy; contempt; admiration/respect; nostalgia; awe/wonder; love). Each annotator codes for each of the 20+ emotions for each social media post. Each annotator is also directed to code separately for their own personal reactions (0 to 100) and the content of the post itself (0 to 100), which includes portrayals and stimuli of each emotion. The absence of emotion is indicated with a zero, whereas 100 indicated the most extremely frequent and/or intense emotion. The numbers in between represent gradients for each emotion. This method allows annotators to make distinctions between closely related emotions (e.g., anger versus hate and contempt; cute/kama muta versus love and admiration), as well as between what is present in the post and what it elicited in them, personally. Given that when the annotation guide was created, cute content and related emotions were not as well defined in the literature or by this research group, this guide offered a heuristic, inclusive set of directions, as follows:

Having juvenile characteristics and evoking positive emotions, such as *kama muta*, which is the heartwarming feeling of seeing something adorable (often occurs with the expression "aww")[1]. Includes the emotion of reacting to cute things.

CuteContent: judge content on the 100-point scale, based on:

1. Attribute
    a. Sensory/appearance characteristics: the degree of neotenic or baby-animal characteristics (baby schema), such as small size, round face, big round eyes, chubby cheeks, dimples, short nose, small chin, large, curved forehead, softness.
    b. Personality characteristics/behavior: clumsy walking, cuddliness, vulnerability, helplessness, curiosity, innocence, playfulness. Behavior can include interaction, which can be either mutual or not reciprocal, and can be between cute entities (e.g., puppies) or a cute entity and a non-cute entity (e.g., a kitten and an adult human).
    c. Could include cute words/exclamations/sounds made by the creature ("eek!") and other non-infant but cute behavior or facial expressions.
    d. Not to be confused with sexual attraction.
2. Cuteness by contrast: sense of cuteness created by contrasting different attributes (e.g., large dog vs. small dog, baby-looking character lifting heavy weights, a wolf in a sheep costume, dog in a tutu).
3. *Kama muta* expressed by the author of a social media post, but <u>only when applied to cute things</u> —"this is so cute!!" counts as indicative of cute content, even if there may not be high levels of attribute or contrast cuteness. Does not include other *kama muta* feelings when not triggered by cute things, such as tears of gratitude, which then would be coded as gratitude (see kama muta lab).
4. If someone refers to an adult human as "cute," and we can't see an image/know what kind of cute, here are some heuristics:
    a. If there's additional verbal information, then go with that (e.g., "cute butt" would go under sexual attraction unless it's a baby's butt that is being discussed).
    b. If we can't tell at all (e.g., "that waiter was cute!") put in zero. If you can tell the target has some juvenile features, say, it's a reference to a specific teenage boy band singer, then you might code it as a 5-15 because we can look up his image and know he has juvenile features. Whereas if we can't see a picture and it's about an adult man or woman, put in a 0).
    c. If there's a reference to something as "cute" and we CAN tell (e.g., the "that's so cute" is a reply to a picture), then rate the "that's so cute!" post based on our interpretation of the poster's perception of the actual image/story/etc. But take into account the poster's intensity of *kama muta*; e.g. "I find that kinda cute" should have a lower score than "OMG that's the cutest thing ever! My heart warms!"
5. If someone refers to a baby or animal as cute and there is no image or additional information, trust that the author is referring to our definition of cute.

Note on scoring: More than one cute entity will receive a higher score than one cute entity. Cute entities interacting will receive a higher score than those not interacting (e.g., multiple cute kittens playing will

---

[1] http://kamamutalab.org/about/ "Kama muta' is Sanskrit for 'moved by love'."

be scored higher than one cute kitten sitting). The more cute traits in the post (e.g., baby schema, cute behavior, interaction, cuteness by contrast, more than one entity), the higher the score. [2]

2 Cuteness Attributes Taxonomy (CAT)

Code	Description	Scale
Cute Human	An image (e.g., photograph, video, drawing, animation, emoji) or a mention of a real human or fictional character (e.g., baby, child, young adult with youthful features). A toy or a figurine depicting a cute human will be annotated with both this code and the Cute Thing code.	0 or 1
Cute Animal	An image (e.g., photograph, video, drawing, animation, emoji) or a mention of a real animal or fictional character (e.g., puppy, kitten). A toy or a figurine depicting a cute animal will be annotated with both this code and the Cute Thing code.	0 or 1
Cute Thing	An image (e.g., photograph, video, drawing, animation, emoji) or a mention of a cute object (e.g., small boat, cute toy, cute outfit, cute bow). The outfit or hat/bow could be on a cute (or non-cute) human or animal, or it could be shown by itself, e.g., on the floor. In order for the outfit/hat/bow to be counted as cute, it needs to be cute by itself, e.g., when on the ground.	0 or 1
Cute Emoji	The use of cute emojis. Refer back to the definition of cute for characteristics and to the list of cute emojis. Example of a cute emoji: 😍	0 or 1
Cute Emoticon	The use of cute text emoticons. Text emoticons are typed using keyboard characters, usually punctuation marks, e.g., :). Refer back to the definition of cute for characteristics and the list of cute emoticons. Example of a cute emoticon: (=^･ω･^=)	0 or 1
Cute Diminutive	The use of diminutives denoting small size, endearment, heartwarming feeling, lovability. Polish examples: milutki, kochaniutki, misiaczek, kotek, koteczek, piesek, bobasek, serduszko, buziaczek. If Cute Diminutive > 0, Cute Talk should automatically be > 0.	0 or 1
Cute Image	An image (e.g., photograph, video, drawing, animation, emoji) of a human, animal, object displaying cute characteristics in appearance. Text (or sound) only = 0, image of any kind = 1. An emoji counts as an image. An emoticon and ASCII text art do not count as an image.	0 or 1
Cute More Than One	An image or a mention of two or more cute humans, animals, things, or a mix or thereof. Examples:<br>Code as More Than One=1: (a) a cute baby in a cute outfit if the outfit is cute by itself, when on the ground; (b) a cute baby with a cute toy (toy needs to be cute by itself); (c) two or more photos of different kittens (one kitten per photo); (d) a photo of a cute puppy and a cute emoji in the text/or the word "cute" in text, but only if the emoji/the word "cute" is referencing something else than the puppy on the photo.	0 or 1

[2] This gif is basically a 100 in cute because it has a baby cat, contrast, interaction, behavior, more than one. https://giphy.com/gifs/cute-aww-eyebleach-lzuJRXR4t2Ep6yRnfl

	Code as More Than One=0: (a) two or more photos of the same cute kitten; (b) a photo of a cute puppy and a cute emoji in the text/or the word "cute" in text, but only if the emoji/the word "cute" is referencing the puppy on the photo.	
Anthropo-morphism	The attribution of human characteristics, emotions, behaviors, to non-human entities (e.g., animals, inanimate objects, abstract concepts). This can be done via visuals or descriptions in text. Examples: animals doing human things (wearing outfits, talking, engaging in uniquely human activities, behaving like humans); objects depicted with human faces, arms or legs; personification of music, winter, liberty. This code will overlap with Cute Contrast. Every post annotated with this code will be annotated with the Cute by Contrast code, but not every post annotated with Cute Contrast code will be annotated with this code.	0 or 1
Baby Schema	A display of baby animal or baby human features, e.g., small size, round face, big round eyes, chubby cheeks, dimples, short nose, small chin, large, curved forehead. 0=no baby schema; 1=several explicit baby schema characteristics.	0 to 100
Cute Talk	Saying cute things, saying them in a cute way (e.g., baby-like). Using high and exaggerated pitch. Using diminutives denoting small size, endearment, heartwarming feeling, lovability. Polish examples: milutki, kochaniutki, misiaczek, kotek, koteczek, piesek, bobasek, serduszko, buziaczek. Using cute words (refer back to the list of cute keywords). If Cute Diminutive > 0, Cute Talk should automatically be > 0.	0 to 100
Cute Interaction	An image or a mention of more than one cute human or animal interacting (e.g., two puppies playing or sleeping together) and amount of interaction from 0 (none) to 100 (lots). To use this code, the interaction needs to be between more than one cute living entities, (e.g., two cute pandas playing together) or between one or more cute living entities and one or more non-cute living entities, e.g., (a kitten and an adult human). Code Interaction=0 for one cute panda playing with a cute toy (but code Behavior as more than zero). Interaction can be either mutual or not reciprocal. Interaction can be either physical or verbal.	0 to 100
Cute Behavior	A human, animal, object displaying cute activities or acts; includes behavior, movement, body posture, facial expressions, sleeping, playing, etc. Interaction should always involve behavior, but behavior will not always involve interaction. This code is not used to annotate wholesome behavior, but sometimes these two codes (Cute Behavior and Wholesome Behavior) might cooccur.	0 to 100
Wholesome Behavior	An act of kindness; we use only the following definition: "an embodiment of the following: self-less, considerate, sweet, compassionate, thoughtful, generous, genuine" (urbandictionary.com). A behavior that makes others feel good, can express love, affection, friendship, familial relationships. Sometimes this code might cooccur with the Cute Behavior code.	0 to 100
Cute Contrast	An image or a mention of a human, animal, object displaying cute characteristics by contrasting attributes (e.g., a small boat next to a large boat, a kitten with its mom, a puppy doing human things such as	0 to 100

	wearing an outfit). 0=no contrast; 100=very sharp and explicit contrast. This code can sometimes overlap with the Anthropomorphism code.

3 Heartwarming Social Media (HSM)

Item	Item	Scale
1	I uttered something like “aww” or “jejku”*	0 to 6
2	I wanted to have this experience again	0 to 6
3	I wanted to tell someone about this experience	0 to 6
4	I wanted to share this experience with others	0 to 6
5	I wanted to touch, hug, pet or play with the entity from this post	0 to 6
6	It was cute	0 to 6
7	It was sweet	0 to 6
8	It was adorable	0 to 6
9	It was delightful	0 to 6
10	It was wholesome	0 to 6

* In Polish, both “aww” and a vernacular “jejku” are common vocalizations when seeing something or someone cute.

4 Details on Data Collection and Processing

We conducted our study in Polish social media and language. Because there is no exact Polish equivalent of the word “cute,” we compiled a list of Polish words commonly used in this sense. First, five native speakers of Polish independently generated lists of words that they believed might be present in Polish tweets with cute content. The combined list contained sixty items, of which seven were generated by more than two speakers. Most items were native Polish words, but the list also contained borrowings from English (cute, słit/sweet, bejbi/baby) and Japanese (kawaii) as well as onomatopoetic strings (e.g., uwu, owo, aww) and letter symbolism (e.g., XOXO). The combined list of sixty words was expanded to include relevant inflectional forms[3] of the inflected (Polish) nouns and adjectives; this process yielded 231 unique keyword strings.

Purposive sampling for cute annotation was performed in multiple stages.

Stage 1. In Stage 1, we used a corpus originally collected for another study involving Polish social media. This corpus contained 762,416 Tweets over a period from July 2009 to January 2020, collected from 303 Twitter accounts identified as influential sociopolitical groups and individuals in Poland.[4] This corpus was searched for Tweets containing at least one of the seven most likely “cute keywords” (i.e., those seven words chosen by more than two Polish speakers). This search was

[3] We included all forms for the Nominative and Genitive cases for all genders and numbers.

[4] For a description of the selection criteria, see Paletz, S. B. F., Johns, M., Murauskaite, E., Golonka, E., Pandža, N. B., Rytting, C. A., Buntain, C., and Ellis, D. *Emotional content and sharing on Facebook: A theory cage match*. Manuscript under review.

further refined by excluding accounts associated with media, politicians, academics, and other professions deemed less likely to share cute content; this process left 117 Twitter accounts searched. The search on these accounts yielded 201 tweets containing at least one of the seven selected keywords in any gender or number, or matching the lemmatized form (as provided by the SpaCy[5] library's Polish lemmatizer). All tweets sampled in this stage were annotated.

Stage 2. Due to the low yield of cute content in the corpus collected in Stage 1, we collected a new corpus of tweets for a more thorough analysis of cute content posted in Polish Twitter. In Stage 2, a new corpus was pulled from Twitter using the original, expanded set of 231 keywords for a five year, seven month period (January 2015 through July 2020) across everyone using Twitter in Poland.[6] This new corpus contained 19,592,791 Tweets across the 67 months. From this new corpus, tweets containing at least two distinct keywords from the list were pulled for the year 2015, limited to one tweet (chosen randomly) per Twitter account, yielding a sample of 3,604 tweets. For the other years in the collection (2016–2020), the sampling was further restricted to tweets containing at least two distinct keywords (as with 2015) *and* some embedded non-text media (e.g., an image or a video). For these years, one tweet meeting the stricter criteria was sampled randomly per Twitter account per year (for those Twitter accounts that had a tweet matching the criteria for that year), yielding 2,431 additional tweets across the five calendar years after 2015.[7] From tweets sampled in this stage, 667 were randomly selected for annotation (the original aim was 600 tweets; 667 is the number of tweets annotators were able to code in the time they had available for this task).

Stage 3. In order to increase the diversity of topics within the annotation, the research team further refined the keywords to include baby animal names and words referring to human babies. The first author, with the team of Polish speaking annotators, generated a list of 40 common baby animal names (e.g., piglet, bunny) and 7 words used to refer to human babies. The list was expanded as before to include inflectional variants; this time only one animal keyword was required in the sampling (along with embedded media files as above). In Stage 3, up to one tweet per Twitter account was sampled for each of the six calendar years represented in the collection (2015–2020), yielding 3,302 tweets. From tweets sampled in this stage, 743 were randomly selected for annotation.

Stage 4. Finally, in order to increase the range or degree of cute content across the annotated set, the collection was augmented with additional 280 tweets randomly selected for annotation out of 2,363 tweets sampled from the same accounts but with no keyword list (Stage 4). For the years 2015, 2016, and 2020, a list was compiled of all those Twitter accounts for which a tweet with cute content had previously been sampled during that year. For those three years, up to four tweets were sampled randomly from each Twitter account in the list for that year, such that none of the keywords were present in the tweets (either from the original sixty keywords or the 47 baby human and animal keywords). No filter was applied on embedded media; i.e., tweets both with and without embedded

---

[5] spacy.io. We used the Polish lemmatizer included in SpaCy version 3.1, which is based on the Morfeusz dictionary (morfeusz.sgjp.pl/en) by the Institute of Computer Science at the Polish Academy of Sciences (PAS).

[6] This new corpus used for Stages 2–4 does not use the same list of sociopolitical influencers as Stage 1, but is open to anyone using Twitter in Poland.

[7] 2020 is treated as a full calendar year for our purposes, even though only seven months of collection were available for it.

media were allowed. (The maximum number of randomly sampled tweets per account per year was increased from one to four because many of the tweets from older years were found to be no longer available; the first author selected the first available tweet from each set of four.) From tweets sampled in this stage, 280 were selected for annotation.

Altogether, 11,901 tweets were sampled in four stages, of which 1,891 were selected for annotation. All tweets sampled in Stage 1 were annotated, while tweets from Stages 2–4 were randomly selected for annotation. Sixteen tweets were unavailable at the time of annotation, resulting in 1,875 tweets annotated.

Utilizing this multi-step methodology to collect and process data allowed us to improve the quality of our sample with each stage and in the end collect a rich sample of posts on diverse topics and containing a range of cute content (on the 0–100 scale) to test new instruments on. However, we acknowledge that this procedure could affect generalizability of our findings to other contexts (see the Limitations section).
